# Hospice preference of the family decision-makers for cancer patients in China: an exploratory study

**DOI:** 10.1186/s12904-022-01112-1

**Published:** 2022-12-15

**Authors:** Nian Yao, Hao Chen, Xiaobin Lai

**Affiliations:** School of Nursing, Fudan University, Shanghai, China

**Keywords:** Hospice enrollment, Cancer, Choice behavior, Decision-making, Physician–patient communication, Chinese population

## Abstract

**Background:**

The reasons for hospice underuse in China need exploration from the perspective of patients with cancer and their families. Furthermore, existing literature about hospice decision-making among Chinese families with cancer patients is limited. This study aimed to investigate the awareness of hospice care among families with cancer patients, their preference for healthcare at the end-of-life stage of care, and the predictors of hospice preference.

**Methods:**

This was an exploratory study conducted between July 2021 and January 2022. Overall, 300 decision-makers of cancer patients were recruited from the oncology ward of seven hospitals in Shanghai, China. Of these, 285 valid responses were included in the data analysis. A self-developed questionnaire about their preference for healthcare when the patient was at the end-of-life stage was completed. Descriptive analysis, t-test, chi-square test, and multivariable logistic regression were conducted to analyze the data.

**Results:**

Only 46.0% of the participants have heard of hospice care. Most participants (78.2%) reported no introduction to hospice care from their doctors. More than half of the participants (58.2%) did not have a preference for healthcare at the end-of-life stage. Seventy-eight (65.5%) of the 119 participants who had a preference chose hospice care, and the other 41 participants (34.5%) refused hospice care. Having heard of hospice care had a significant impact on preferring healthcare at the end-of-life stage (adjusted *OR* = 14.346, 95%CI 7.219–28.509, *p* < 0.001). Not being sure whether the doctor introduced hospice care before had a significant impact on having no preference for healthcare at the end-of-life stage (adjusted *OR* = 0.180, 95%CI 0.052–0.617, *p* = 0.006). Another family member being cared for at home had a significant impact on the participants’ hospice preference (adjusted *OR* = 2.739, 95%CI 1.159–6.470, *p* = 0.022).

**Conclusion:**

The end-of-life communication between healthcare providers and the families of cancer patients is insufficient. More efforts should be made in increasing the awareness of hospice care among patients with cancer and their families. Further study is needed to explore the reasons for a lack of discussion on hospice options between healthcare providers and the patients’ families. Additionally, the impact of the at-home care burden on the hospice choice of families with cancer patients requires further study.

**Supplementary Information:**

The online version contains supplementary material available at 10.1186/s12904-022-01112-1.

## Background

Cancer is a major public health problem in China. It was estimated that 4,064,000 new cancer cases and 2,413,500 cancer deaths occurred in China in 2016 [1], which was higher compared to previous years [2, 3]. Thus, the cancer burden in China has been increasing. In this regard, the cancer population needing hospice care is also increasing. Hospice care is essential for patients at the end-of-life (EOL) stage to have good dying and death quality. Yet, a large number of patients with late-stage illness did not receive hospice care before their death. According to the World Health Organization, only about 14% of people who need palliative care currently receive it [4]. The situation in China was similar, whereby it was estimated that only 0.3% of people who needed hospice care received the service [5]. An important reason for this is lack of hospice services in China. As a result, establishing hospice services has been put on the agenda of the National Health Committee. Consequently, since 2017, 71 areas in China were chosen as the pilot settings for hospice care [6].

Localized hospice services are recommended because of the variety of the client population, socio-cultural background, and economic level in the pilot areas. Healthcare institutions are involved in providing hospice service at three levels in the pilot areas, that is, community health service centers, secondary hospitals, and tertiary hospitals. Despite the variety of healthcare institutions, hospice services are usually provided in outpatient settings, inpatient settings, and at patients’ homes. Inpatient hospice service is the main service mode. The patients are cared for in the hospice ward of a healthcare institution by the hospice care team. At-home hospice services are usually provided by doctors and nurses. Hospice services in the outpatient settings are an important part of the entire hospice care system. The family members of a patient who may need hospice care can consult with hospice doctors in the outpatient settings. The doctors can examine the patient’s condition and triage them to inpatient or at-home hospice service, if and as needed.

Despite the increasing availability of hospice services, the underuse of the service may be another difficulty in China [7]. That is, increasing hospice services does not equate to more benefits for patients who need hospice care. However, only few studies have examined the utilization of hospice services in China. Studies that did examine this mainly focused on the hospice care in Shanghai, where hospice care has been provided in all community health service centers (i.e., primary healthcare institutions) since 2020. According to Wang et al.’s study, the utilization rates of hospice beds in 10 of the 16 districts of the city were higher than 50% [8]. The lowest utilization rate among the 16 districts was less than 20% [8]. Similar findings were echoed in other studies [9, 10]. Thus, the utilization of hospice care is unsatisfactory. Promoting the use of hospice services could become another important task for healthcare providers and policymakers in China in the future.

The reasons for hospice underuse are multifactorial. A local study found that the difficulty of the hospice service, from the perspective of hospice care providers, arose from the aspects of patients and their families, healthcare providers, the healthcare system, and policy and regulations [11]. Hospice care providers suggested that the unawareness and unacceptance of patients and their families regarding hospice care were two key factors impeding their use of such care [11]. However, this must be further analyzed from the perspective of the patients and their families. Whether patients should receive hospice care is a very difficult decision for families of patients at the EOL stage. Patients and their families face elevated emotional distress in choosing between intensive treatment with potential side effects, or less aggressive treatment to maximize the quality of life [12]. Previous foreign studies found that patients’ conditions, values, and family-related factors contributed to the EOL stage treatment decision [13–18].

Existing literature has focused on EOL stage decision-making to some extent [19]. Two studies analyzed the implementation of resuscitation among deceased patients with cancer retrospectively [20, 21]. A qualitative study explored the factors associated with family members’ medical decision-making in the EOL stage of advanced cancer patients [22]. Wang et al. found in a qualitative study that most families still chose non-invasive resuscitation treatment for patients receiving hospice care in the community of Beijing [23]. Moreover, a few studies examined hospice preference and choice among the Chinese population. A quantitative study reported that the acceptance of hospice care among the community residents in Hebei province was not high and their preference for hospice care was significantly influenced by older age and a higher education background [24]. A previous study explored the reasons why dying patients and their families chose a hospice ward and found that the availability of being hospitalized was the main reason for their choice [25].

Existing literature about EOL stage decision-making among Chinese patients and their families is limited. Therefore, further research on hospice decision-making among the Chinese population is needed. This study, thus, aimed to investigate the family members’ awareness of hospice care, who make medical decisions for the cancer patients, their preferences for healthcare at the EOL stage, and the predictors of their choice of hospice care.

## Methods

### Design and setting

This was an exploratory study with a convenience sample recruited in the oncology ward of four tertiary hospitals and three secondary hospitals in Shanghai, China.

### Participants

The study was conducted between July 2021 and January 2022. The inclusion criteria for the participants were as follows: (i) being a family member of a patient diagnosed with cancer and the patient being over 18 years old; (ii) being the primary medical decision-maker for the patient. In the family-dominated society in China, the medical decisions of a patient with cancer were usually made by the family members even when the patient had the capacity to make decisions. When a cancer patient was hospitalized, the doctor would ask the family to designate at least one family member to be the “primary decision-maker.” The person would be responsible for the medical decisions and for signing medical documents for the patient; and (iii) being able to operate a mobile phone. The exclusion criteria were as follows: (i) not being a family member of the patient and (ii) being unable to operate a mobile phone. One staff member in each ward was designated to recruit participants and collect the data. The designated staff member explained the study to the eligible family members of the patients in a face-to-face format and invited them to participate in the study.

### Instruments and data collection

An online questionnaire was developed by the researchers for data collection. The questionnaire was developed based on previous literature [16, 25, 26] and discussed within the research team. Some revisions were made after two experienced nurses working in a palliative ward and in an oncology ward, respectively, reviewed the questionnaire. Afterward, the printed questionnaire was piloted among the primary decision-makers of five cancer patients and was transferred into the online version after the pilot study.

The questionnaire consisted of four parts. The first part was information about the participants (i.e., the primary decision-makers of the cancer patients), including their demographic characteristics, the person making medical decisions, their perception of the patient’s quality of life (QOL), their satisfaction with the patient’s QOL, and their perception of the patient’s disease progression. The person making medical decisions was asked using a single question, “who made medical decisions for the patient?” The answer categories included patient per se, patient’s spouse, patient’s child, patient’s sibling, and others. The participants’ perception of the patients’ QOL was rated on a 1–5 Likert scale (1 = no quality; 5 = very high quality). Their satisfaction with the patients’ QOL was also rated on a 1–5 Likert scale (1 = totally dissatisfied; 5 = totally satisfied). The participants’ perceptions of the disease’s progression were classified into five categories (i.e., being cured, becoming better, no change, deteriorating, and at the terminal stage). Questions regarding whether they have heard of hospice care and whether the doctors had introduced hospice care were also asked.

The second part of the questionnaire was about the cancer patients for whom the participants made medical decisions. The information about the patients included their demographic characteristics, the cancer duration, metastatic status of the cancer, treatment type, past health history, their Palliative Performance Scale (PPS) score, and the patient’s perception of the disease progression. Since the PPS was not adopted in the routine clinical practice of the seven wards in which the data were collected, the items of PPS were separated into single questions, with multiple choices for the participants to choose from. For example, regarding the item of “ambulation” in the PPS, the participants were asked, “please tell me the patient’s status of ambulation during these days?” The answer categories included “full”, “reduced”, “mainly sit or lie”, and “totally bed bound”. The final PPS scores of all patients were judged by one of the researchers (X.L.) based on the participants’ responses. The palliative nurse who reviewed the questionnaire helped train the researcher to use the PPS before the researcher judged the PPS scores. The dividing line of 60% was adopted, in accordance with the validated Chinese versions of PPS [27]. The content validity index of the Chinese version ranged from 0.83 to 1.00. The value of factor loading was higher than 0.86. The Cronbach’s α was 0.97. The criterion validity with KPS was 0.92 [27].

The third part was about family caregiving, which included information about the type of primary caregiver, age of the primary caregiver, working status of the primary caregiver, time spent on caregiving per day, another one in need of being cared for at home, subjective health status, and subjective care burden. The working status of the caregiver was classified into five categories, which were the same as that of the participants’ working status, including retired, full-time job with frequent absence, full-time job with no absence, part-time job, and others. “Frequent absence” was defined as being absent from work more than two times a week to care for the patient. The subjective health status of the caregiver was rated on a 1–5 Likert scale (1 = very poor; 5 = very good). The item “another one in need of being cared for at home” was to enquire whether the primary caregivers had anyone else they needed to care for at home (i.e., parents, children, grandchildren, and others). The subjective care burden was also rated on a 1–5 Likert scale (1 = no burden; 5 = very high burden).

The fourth part was the participant’s preference for healthcare at the EOL stage. The participants were asked whether they would choose hospice care for the patient when the patient was at the EOL stage. Six answer categories were provided, including “yes,” “no,” “unable to decide,” “never thought about it,” “not aware of hospice care,” and “refuse to answer this question.” A participant was defined as “having a clear preference” if the participant chose “yes” or “no” when asked about this preference. A participant was defined as “having no clear preference” if the participant chose any of the other four options. They were then asked to provide the reasons for their selection via multiple-choice questions. The full questionnaire could see Additional file 1.

The participants in the main study completed the online questionnaire using their own mobile phones. They could ask the designated staff members for assistance if they had difficulties understanding the questions. They made their choices on the phone after explanations from the staff members. The designated staff members were trained by the researcher (X.B.L.) before the study began. Overall, 300 questionnaires were distributed and 285 effective questionnaires (95%) were included for data analysis.

### Data analysis

SPSS version 20.0 was used for data analysis. Descriptive statistics were reported as frequencies, means, standard deviations (SDs), median, minimum, maximum, and percentages. The differences between the groups were obtained using the T-test and Pearson’s Chi-square test, including the differences between the groups with and without a clear preference for healthcare at the EOL stage and the group preferring hospice and the group refusing hospice care, respectively. Then the associations between the two dependent variables (i.e., the clear preference for healthcare at the EOL stage and the preference for hospice care) and the aforementioned independent variables were quantified using multivariable logistic regression models, respectively. A *p*-value of less than 0.05 was considered statistically significant.

### Ethical considerations

The study received approval from the ethics committee of the school in which the authors worked (Approval number: IRB#2022–09-7). The study began after we received approval from the school in which the authors worked and from the managers of the seven oncology wards. The designated staff members informed the primary decision-makers of the patients about the study’s objective and provided detailed information about the study when they were invited to participate. The principles of voluntary participation and confidentiality were ensured for the participants. The informed consent form functioned as the first page of the online questionnaire. The participant clicked the “agree” button, confirming that they agreed to participate before they continued completing the questionnaire. All methods were conducted in accordance with the ethical standards of the declaration of Helsinki.

## Results

### Information about the participants

Overall, 300 family members who were the primary decision-makers of cancer patients were recruited. Of these, 285 valid responses were included in the data analysis. Owing to the fact that three patients were under the age of 18, eight patients’ diagnoses had not yet been confirmed, and four patients were transferred to hospice wards in community health service centers as a result of their responses, data from 15 decision-makers were excluded from the analysis. It was probable that the decision-makers of the four transferred patients were invited when these patients were in the study hospitals. However, the patients were soon discharged and admitted to the hospice ward in the community health service centers. The decision-maker finished the questionnaire after the patient was discharged. The mean age of the participants (i.e., the medical decision-maker of the patients) was 53.47 years old (*SD*: 13.38). The most common decision-makers were the patients’ children (38.6%, *n* = 110). Most participants had finished middle/high school (49.8%, *n* = 142) or college/university (38.2%, *n* = 109). Approximately half of the participants were retired (42.6%, *n* = 121). For the participants who had a full-time job, more than half (53.3%, *n* = 57) frequently asked for leave. Half of the participants (50.5%, *n* = 144) thought the patient’s QOL was at a moderate level and most (71.6%, *n* = 204) were satisfied with the patient’s QOL. There were 15.8% (*n* = 45) of the participants who thought the patient was deteriorating and 12.3% (*n* = 35) who thought that the patient was at the terminal stage. Only 46.0% (*n* = 131) of the participants had heard of hospice care, with fewer having heard of hospice care from doctors (14.4%, *n* = 41). Most participants (78.2%, *n* = 223) reported that the doctors did not introduce hospice care to them. Detailed information about the participants is presented in Table 1.

**Table 1:** The demographic information about the participants and their perception of the patients’ condition (*N* = 285)

Variables	*Mean*	SD
**Age**	53.47	13.38
	***n***	**%**
**The person making medical decisions**		
Patient’s child	110	38.6
Patient	71	24.9
Patient spouse	87	30.5
Patient’s parents/siblings/grandchildren	17	6.0
**Educational background of the participants**		
Primary school (6 years of schooling)	20	7.1
Middle/high school (9–12 years)	142	49.8
College/University (15–17 years)	109	38.2
Postgraduate (at least 18 years)	14	4.9
**Working status of participants**		
Retired	121	42.6
Full-time job, frequent absence	57	19.7
Full-time job, no absence	50	17.6
Part-time job	21	7.4
Other (unemployed/freelance)	36	12.7
**Participants’ perception of the patients’ QOL**		
No quality	36	12.7
Poor quality	56	19.6
Moderate quality	144	50.5
High/very high quality	49	17.2
**Participants’ satisfaction with the patients’ QOL**		
Totally dissatisfied	38	13.3
Very dissatisfied	43	15.1
Satisfied	124	43.5
Very satisfied/Totally satisfied	80	28.1
**Participants’ perception of the disease progression**		
Being cured	53	18.6
Becoming better	97	34.0
No change	55	19.3
Deteriorating	45	15.8
At terminal stage	35	12.3
**Having heard of hospice care**		
Yes	131	46.0
No	154	54.0
**Doctor introduced hospice care before**		
Yes	41	14.4
No	223	78.2
Not sure	21	7.4

*QOL* Quality of life

### Information about the patients and family caregiving

The mean age of 285 patients was 64.09 years old (*SD* = 12.32). More than half (58.2%, *n* = 166) were male. Most of the patients were married (87.0%, *n* = 248), educated at middle/high school level (59.6%, *n* = 170), and without a religious belief (84.9%, *n* = 242). The median duration of the cancer was one year (min = 0.1y; max = 34y). Less than half of the patients had distant metastasis (47.7%, *n* = 136) and were aware of their diagnosis and severity (48.4%, *n* = 138). The PPS score of 55.8% of the patients (*n* = 159) was higher than 60%. The most common treatment they were undergoing was chemotherapy (54.0%, *n* = 154), targeted therapy (16.5%, *n* = 47), and immunotherapy (14.4%, *n* = 41). Most patients (96.5%, *n* = 275) had non-cancer chronic conditions. The detailed characteristics of the patients are presented in Table 2.

**Table 2:** Demographic and disease-related characteristics of the patients (*N* = 285)

**Variables**		***Mean***	**SD**
**Age**		64.09	12.32
		***n***	**%**
**Gender**	Female	119	41.8
	Male	166	58.2
**Marital status**	Married	248	87.0
	Widowed	24	8.4
	Unmarried	8	2.8
	Divorced	4	1.4
	Cohabited	1	0.4
**Educational background**	Primary school (6 years of schooling)	49	17.2
	Middle/high school (8–12 years)	170	59.6
	College/University (15–17 years)	58	20.4
	Postgraduate (at least 18 years)	8	2.8
**Religion**	No	242	84.9
	Yes	43	15.1
**Payment method of medical expense**	Basic medical insurance for urban residents	131	46.0
	Basic medical insurance for urban employees	98	34.4
	New rural cooperative medical insurance	45	15.8
	Self-financed/Commercial insurance	11	3.8
**Monthly disposable income per capita**	< 2500RMB	55	19.3
	2500–5000RMB	92	32.3
	5000–10000RMB	107	37.5
	> 10,000–15000RMB	31	10.9
**Distance metastasis**	Yes	136	47.7
	No	149	52.3
**PPS score**	< = 60%	126	44.2
	> 60%	159	55.8
**Undergoing treatment**	Chemotherapy	154	54.0
	Target therapy	47	16.5
	Immunotherapy	41	14.4
	Surgery	33	11.6
	Radiotherapy	14	4.9
	Hormone therapy	9	3.2
	Interventional therapy	10	3.5
**With non-cancer chronic conditions**	Yes	275	96.5
	No	10	3.5
**Patients’ perception of the disease progression**	Did not know	24	8.4
	Only knew the diagnosis	123	43.2
	Knew the diagnosis and the severity	138	48.4

Most patients (96.9%, *n* = 276) had a family member as the primary caregiver during their hospitalization. A few families (16.5%, *n* = 47) also hired a nursing assistant for the patient. The mean age of the primary caregivers was 56.78 years old (*SD* = 12.55). More than half of the primary caregivers were the patients’ spouses (59.3%, *n* = 169) and were retired (55.5%, *n* = 158). The median length of caring for the patient in the hospitals was 24 h per day. Approximately half of the families (46.0%, *n* = 131) had another one to be cared for at home. Most participants thought the care burden was at a moderate (34.2%) or high (34.2%) level (see Table 3).

**Table 3:** Information about family caregiving (*N* = 285)

Variables	*n*	%
**Primary caregiver**		
Spouse	169	59.3
Children	80	28.1
Parents	13	4.6
Siblings	12	4.2
Grandchildren	2	0.7
Others (domestic helper/nursing assistant)	9	3.1
**Working status of primary caregivers**		
Retired	158	55.5
Full-time job, frequent absence	53	18.6
Part-time job	22	7.7
Full-time job, no absence	20	7.0
Others (unemployed/freelance)	32	11.2
**Subjective health status of primary caregivers**		
Very poor/Poor	32	11.3
Moderate	124	43.5
Good	85	29.8
Very good	44	15.4
**Another one in need of being cared for at home**		
Nobody	154	54.0
Parents	64	22.5
Children	48	16.8
Grandchildren/others	19	6.7
**Subjective care burden**		
No burden/low burden	50	17.6
Moderate burden	97	34.0
High burden	98	34.4
Very high burden	40	14.0

### Healthcare preference at the EOL stage

When asking their preference for healthcare at the EOL stage, 58.2% of the participants (*n* = 166) did not have a clear preference. Seventy-one participants did not have a clear preference because they were not aware of hospice care (42.8%), sixty-eight participants (41.0%) never thought about the care plan at the EOL stage, fourteen were unable to decide (8.4%), and thirteen refused to answer (7.8%). Among the 119 participants who had a clear preference, there were 78 participants (65.5%) who chose hospice care and 41 participants (34.5%) who refused hospice care. The reasons for preferring hospice care were relieving suffering (85.9%, *n* = 67), better QOL (60.9%, *n* = 47), avoiding unnecessary treatment (35.9%, *n* = 28), and the patient’s will (4.7%, *n* = 4). The reasons for refusing hospice care were saving the patient’s life undoubtfully (70.7%, *n* = 29), not knowing of the service (22.0%, *n* = 9), fulfilling the filial piety (9.8%, *n* = 4), and hospice care being unhelpful (2.4%, *n* = 1).

### Associated factors with the participants’ clear preference

There were significant differences in a few variables between the participants with a preference (*n* = 119) and those without a preference (*n* = 166) (see Table 4), including the age of primary caregivers (*t* = 1.97, *p* = 0.05), working status of the decision-makers (*χ*^2^ = 9.82, *p* = 0.04), having heard of hospice care (*χ*^2^ = 80.53, *p* < 0.00), and doctors’ introduction of hospice care (*χ*^2^ = 20.95, *p* < 0.00). The participants with a clear preference (Mean = 58.49, SD = 11.68) were significantly older than those with no clear preference (Mean = 55.50, SD = 13.05). The proportion of retired participants was significantly higher in the group with a clear preference (52.1%) than in the group with no clear preference (35.5%). More participants in the group with a clear preference (77.3%) have heard of hospice care before and have heard of hospice care from doctors (25.2%) than those in the group with no clear preference (23.5%; 6.6%). No significant differences were found on the other variables of the patients, family caregiving, and primary decision-makers between the two groups.

**Table 4:** The differences between the participants with clear preference and with no clear preference (*N* = 285)

	**With clear Preference **^**#**^ **(*n* = 119)**	**With no clear** **preference** **(*n* = 166)**	**t-statistic**	**Degrees of freedom**	***p*-value**
	Mean (SD)	Mean (SD)			
**Age of primary caregivers**	58.49 (11.68)	55.50 (13.05)	1.97		0.05
	n (%)	n (%)	**Chi-square statistic**	**Degrees of freedom**	***p*-value**
**Working status of participants**			9.82	4	0.04
Retired	62 (52.1)	59 (35.5)*			
Part-time job	10 (8.4)	11 (6.6)			
Full-time job	17 (14.3)	33 (19.9)			
Absent from duty frequently	17 (14.3)	40 (24.1)*			
Unemployed/freelance	13 (10.9)	23 (13.9)			
**Having heard of hospice care**			80.83	1	< 0.00
Yes	92 (77.3)	39 (23.5)			
No	27 (22.7)	127 (76.5)			
**Hospice introduction from doctors**			20.95	2	< 0.00
Yes	30 (25.2)	11 (6.6)*			
No	84 (70.6)	139 (83.8)*			
Not sure	5 (4.2)	16 (9.6)			

Note. ^#^A participant was defined as “having a clear preference” if the participant chose “yes” or “no” when asking the preference for healthcare at the EOL stage. A participant was defined as “with no clear preference” if the participant chose any of the other four options. *The group with a clear preference was significantly different from the group without a clear preference (*p* < 0.05)

The multivariable logistic regression analysis suggested significant associations of both having heard of hospice care and doctors’ introduction of hospice care, with the preference for healthcare at the EOL stage. Participants who had heard of hospice care were 13.35 times more likely to have a clear preference than those who have not heard of hospice care (adjusted OR = 14.35, 95%CI 7.22–28.51, *p* < 0.01). Being unsure whether the doctor had introduced the hospice care was significantly associated with having no preference for healthcare at the EOL stage of care (adjusted *OR* = 0.18, 95%CI 0.05–0.62, *p* = 0.01) (see Table 5).

**Table 5:** The multivariable logistic regression analysis of the clear preference for healthcare at the EOL stage (*N* = 285)

Independent variable	B	Wald	Degrees of freedom	Adjusted OR	*p*	95%(CI)	
						**Low**	**High**
**Heard of hospice care**	2.66	57.78	1	14.35	0.00	7.22	28.51
**Age of primary caregivers**	0.02	1.67	1	1.02	0.20	0.99	1.05
**Doctors’ introduction of hospice care**							
No (reference category)							
Not Sure	-1.72	7.45	1	0.18	0.01	0.05	0.62
Yes	-0.19	0.05	1	0.90	0.85	0.37	2.27
**Working status of participants**							
Retired (reference category)							
Part-time job	-0.05	0.01	1	0.95	0.94	0.28	3.22
Full-time job	-0.81	3.42	1	0.45	0.07	0.19	1.05
Absent from duty frequently	-0.65	2.13	1	0.52	0.14	0.22	1.25
Unemployed/freelance	-0.78	2.35	1	0.46	0.13	0.17	1.24
**Constant**	-2.13	5.54	1	0.12	0.02		

### Associated factors with the participants’ preference for hospice care

Among the 119 participants with a preference for healthcare at the EOL stage, the differences between those preferring hospice care (*n* = 78) and those refusing it (*n* = 41), were compared. Seven variables were significantly associated with the participants’ preference for hospice care, including hiring a nursing assistant (*χ*^2^ = 5.79, *p* = 0.02), having another family member being cared for at home (*χ*^2^ = 4.17, *p* = 0.04), participant’s perception of the patient’s QOL (*χ*^2^ = 8.07, *p* = 0.05), participant’s satisfaction with the patient’s QOL (*χ*^2^ = 8.21, *p* = 0.04), participant’s perception of the disease progression (*χ*^2^ = 11.00, *p* = 0.01), having heard of hospice care (*χ*^2^ = 66.44, *p* < 0.00), and doctor’s introduction of hospice care (*χ*^2^ = 6.57, *p* = 0.03) (see Table 6). The participants preferring hospice care hired a nursing assistant (25.6%) more than those refusing hospice care (7.3%). Additionally, more participants preferring hospice care need care for another family member at home (48.7%) than those refusing hospice care (29.3%). The participants refusing hospice care had a higher perception of the patient’s QOL and higher satisfaction with the patient’s QOL. More participants preferring hospice care (37.2%) could understand that the patient’s health was deteriorating or was at the terminal stage than those refusing hospice care (12.2%). No significant relationships were found between other variables and the participants’ preferences (Table 6).

**Table 6:** The differences between the participants preferring hospice care and refusing hospice care (*N* = 119)

	**Preferring hospice care (*n* = 78)** n (%)	**Refusing hospice care (*n* = 41)** n (%)	**Chi-square statistic**	**Degrees of freedom**	***p*-value**
**Hiring a nursing assistant**			5.79	1	0.02
Yes	20 (25.6)	3 (7.3)			
No	58 (74.4)	38 (92.7)			
**Another one in need of being cared for at home**			4.17	1	0.04
Yes	38 (48.7)	12 (29.3)			
No	40 (51.3)	29 (70.7)			
**Participants’ perception of the patients’ QOL**			8.07	3	0.05
No quality	14 (17.9)	6 (14.6)			
Poor	18 (23.1)	3 (7.3)*			
Moderate	25 (32.1)	23 (56.1)*			
High/very high	21 (26.9)	9 (22.0)			
**Participants’ satisfaction with the patients’ QOL**			8.21	3	0.04
No satisfaction	14 (17.9)	6 (14.6)			
low satisfaction	15 (19.2)	1 (2.4)*			
Moderate satisfaction	24 (30.8)	20 (48.8)			
Satisfactory/Very satisfactory	25 (32.1)	14 (34.2)			
**Participants’ perception of the disease progression**			11.00	3	0.01
Being cured	10 (12.8)	10 (24.4)			
Becoming better	29 (37.2)	15 (36.6)			
No change	10 (12.8)	11 (26.8)			
Deteriorating/Terminal stage	29 (37.2)	5 (12.2)*			
**Having heard of hospice care**			66.44	1	< 0.00
Yes	78 (0)	14 (34.1)			
No	0 (0)	27 (65.9)			
**Hospice introduction from doctors**			6.57	2	0.03
Yes	25 (32.1)	5 (12.2)*			
Not sure	4 (5.1)	1 (2.4)			
No	49 (62.8)	35 (85.4)*			

^*^The group preferring hospice care was significantly different from the group refusing hospice care (*p* < 0.05)

Moreover, the multivariable logistic regression analysis suggested a significant association between having another family member being cared for at home, with the participants’ preference for hospice care. Participants who had another family member being cared for at home were 1.74 times more likely to prefer hospice care than those who had no one being cared for at home (adjusted *OR* = 2.74, 95%CI 1.16–6.47, *p* = 0.02) (see Table 7).

**Table 7:** The multivariable logistic regression analysis of the preference for hospice care (*N* = 119)

Independent variable	B	Wald	Degrees of freedom	Adjusted OR	*p*	95%(CI)	
						**Low**	**High**
**Another one being cared for at home**	1.010	5.28	1	2.74	0.02	1.16	6.47
**Hiring a nursing assistant**	0.79	0.93	1	2.20	0.34	0.44	10.97
**Participants’ perception of patients’ QOL**							
High/very high (reference category)							
Moderate	-1.23	2.65	1	0.29	0.10	0.07	1.29
Poor	-0.42	0.15	1	0.66	0.70	0.08	5.52
No quality	-2.32	3.63	1	0.10	0.06	0.01	1.07
**Participants’ satisfaction with patients’ QOL**							
Satisfactory/very satisfactory (reference category)							
Moderate satisfaction	0.10	0.02	1	1.11	0.89	0.27	4.51
Low satisfaction	1.68	1.56	1	5.37	0.21	0.38	75.35
No satisfaction	0.34	0.11	1	1.40	0.74	0.20	9.99
**Participants’ perception of disease progression**							
Being cured (reference category)							
Becoming better	0.72	1.29	1	2.05	0.26	0.60	7.03
No change	0.16	0.05	1	1.18	0.83	0.28	5.01
Deteriorating/terminal stage	1.91	3.31	1	6.72	0.07	0.86	52.42
**Hospice introduction from doctors**							
No (reference category)							
Not sure	0.23	0.03	1	1.26	0.86	0.09	17.59
Yes	1.14	3.22	1	3.14	0.07	0.90	10.97
**Constant**	0.02	0.00	1	1.02	0.97		

## Discussion

In the study, only 41.8% of the decision-makers had a preference for healthcare at the EOL stage of care. Among them, 41.0% never thought about the healthcare plan at the EOL stage of care. In addition, no differences were found in the PPS level and the metastatic status of the patients between the group with and without a preference. The findings indicate that cancer patients and their families may not consider the healthcare option at the EOL stage even when the patient is at the advanced stage of cancer. EOL stage communication between the healthcare providers and the patients and their families is critical to initiate the family’s consideration of their EOL stage care plan [28, 29]. However, inadequate communication about prognosis, treatment choices, and healthcare options is common among patients with an advanced illness [30, 31], which contributes to unrealistic expectations of the patients and their families regarding curability and delayed enrollment in hospice care [28, 32, 33].

EOL stage communication between healthcare providers and the families of patients at the EOL stage in China was under-reported. In this study, 46% of the decision-makers had heard of hospice care but only 14.4% reported that their doctors introduced hospice care before. A previous study reported that social media and friends and relatives were the main information sources of families with dying patients about hospice care in Shanghai; only 17.1% of the families knew about hospice care from their previous doctors [25]. These findings suggest that Chinese healthcare providers’ discussions about the option of hospice care with patients with advanced illness and their families may not be common. In fact, the information needs of family caregivers of patients with a late-stage illness are identified in the literature. Waldrop et al. found that family caregivers of patients with late-stage cancer wanted information about symptom management as well as hospice and palliative care [34]. Families need communication with their healthcare providers to help them understand the progression of the patients, what to expect, and available options for care [34]. Good EOL stage communication could facilitate the hospice decision of patients and their families [28]. A greater exploration of EOL stage communication between healthcare providers and families with cancer patients in China is thus needed in the future.

In this study, two variables (i.e., having heard of hospice care and doctors’ introduction of hospice care) predicted having a preference for healthcare at the EOL stage. Some of the decision-makers did not make a choice because they did not know about the service. The findings indicate that information about the availability of hospice care and the philosophy of hospice care could encourage decision-makers to consider the healthcare at the EOL stage in advance. Similarly, Bazagran et al. revealed that the family’s engagement in EOL stage decision-making was associated with higher awareness of palliative care [35]. Wicher et al. summarized that the knowledge of hospice care would influence its preference at the EOL stage among African Americans [36]. However, the lack of understanding of hospice care is pervasive among patients and their families [34, 37–42]. Therefore, more efforts should be made in increasing the awareness of hospice care among patients with life-threatening illnesses and their families.

This study found that the decision-makers who had others to care for at home had a higher possibility of preferring hospice care for the patients. “another one in need of being cared for at home” refers to a parent with a chronic condition, the elderly, or children under 18 years old. The finding suggests that the total care burden of the family could be a key factor in predicting the hospice decision. That is, caring for cancer patients is emotionally, socially, physically, and financially challenging for family caregivers [43]. Family caregivers experience increasing care burden with the patient’s disease progression [44]. Similarly, the family’s care burden can further increase when another family member must be cared for at home simultaneously. This challenges the family’s resources regarding their capacity for care. The decision-makers may balance the situation and make a choice that is best for the whole family. However, this study only compared the group having a family member to be cared for at home and the group having no member to be cared for at home. Further study is thus needed to identify the impact of caring for different family members at home on the total family care burden, which further influences the hospice decision.

### Limitations

A few limitations should be noted when generalizing the findings of this study. The sample size of the decision-makers who had a preference was small. Larger sample size is thus needed in future studies to identify the characteristics of families with hospice preferences. Another limitation is that the study was conducted in one metropolis located in the east region of China. Thus, these findings are limited regarding generalization to other regions in China, especially for people residing in small towns and the countryside. However, this study’s findings can be referenced by healthcare providers and policymakers in other big cities sharing a similar social-cultural background. EOL stage care preference of patients with life-threatening illnesses in the middle and western regions of China still needs more exploration. One regrettable aspect of the study was that patients were not recruited to avoid potentially disclosing the truth to them, which could violate the family’s request, since hiding the truth is very common in the families of cancer patients in China. A promising result is that a small proportion of the patients made their own medical decisions. Hence, the preference for healthcare at the EOL stage should be explored from the perspective of the patients in the future. Additionally, EOL stage communication between healthcare providers and families with patients at the advanced stage of cancer in China needs to be further empirically investigated.

## Conclusion

A certain proportion of the decision-makers of patients with cancer do not have a preference for healthcare at the EOL stage. The awareness and introduction of hospice care from doctors could promote decision-makers to consider the healthcare option at the EOL stage. Among the decision-makers who have a preference, most of them prefer hospice care when the patient is at the EOL stage. However, when there is another family member being cared for at home, the decision-maker is more likely to prefer hospice care. Thus, this study’s findings provide important implications for healthcare providers on more effective communication regarding hospice care and considering the burden of care of families of cancer patients. These findings also provide a basis for additional studies on EOL stage communication and hospice decision-making among the Chinese population in the future.

## Supplementary Information

**Additional file 1.** The questionnaire of hospice preference of the family decision-makers for cancer patients. The file is the questionnaire the research team developed for the study.

## Data Availability

The datasets generated during the current study are not publicly available because the data were in Chinese characters but are available from the corresponding author at a reasonable request.

## Abbreviations

EOL: End-of-life
QOL: Quality of life
PPS: Palliative Performance Scale
SD: Standard deviation
