# Supplementary material for: Hospice preference of the family decision-makers for cancer patients in China: an exploratory study
Source: BMC Palliat Care. 2022 Dec 15;21:222. doi: 10.1186/s12904-022-01112-1 (PMC9753404; doi:10.1186/s12904-022-01112-1)
Supplement: Supplementary file 1 — Additional file 1. The questionnaire of hospice preference of the family decision-makers for cancer patients. The file is the questionnaire the research team developed for the study. [file 12904_2022_1112_MOESM1_ESM.docx]

**The questionnaire on hospice preference of the family decision-makers for cancer patients**

**Note**: The original questionnaire is in Chinese. You may contact the author for the original version of the questionnaire.

**Instruction**: The following questions are about how you and your family members care for the patient and make medical decisions for the patients. Please choose the most appropriate items which fit your situation best.

**Part I: Information about the primary decision-maker**

1 Your age: ________

2 Your education background:

□Primary school □Middle/high school □College/University □Postgraduate

3 Your working status currently:

□Full-time job with no absence □Full-time job with frequent absence

□Part-time job □Retired □Other, please specify:_________

4 How do you think of the patient’s quality of life?

□1=No quality □2=Poor quality □3=Moderate quality

□4=High quality □5=Very high quality

5 Are you satisfied with the patient’s quality of life?

□1=Totally dissatisfied □2=Very dissatisfied □3=Satisfied

□4=Very satisfied □5=Totally satisfied

6 How do you think of the patient’s disease progression?

□Being cured □Becoming better □No change □Deteriorating □At terminal stage

7 Have you ever heard of hospice care? □ Yes □No

8 Has the patient’s doctor introduced the hospice care before? □ Yes □No □Not sure

9 Who made the medical decisions for the patient?

□Patient per se □Patient’s spouse □ Patient’s child

□Patient’s siblings □Other, please specify:______

**Part II: Information about the patient**

1 The patient’s age: ________

2 The patient’s gender: □Male □Female

3 The marital status of the patient:

□Married □Widowed □Unmarried □Divorced □Cohabited

4 The education background of the patient:

□Primary school □Middle/high school □College/University □Postgraduate

5 Does the patient have a religious belief? □No □Yes, please specify__________

6 How is the patient’s medical expense paid?

□Basic medical insurance for urban residents □Basic medical insurance for urban employees

□New rural cooperative medical insurance □Commercial insurance

□Self-financed

7 Monthly disposable income per capita in the family：

□2500–5000RMB □5000–7500RMB

□7500–10000RMB □>10000–15000RMB

8 How long has it been since the patient was diagnosed with cancer the first time? ________years

9 Does the patient have distance metastasis now? □No □Yes

10 What kind of treatment is the patient undergoing now (you may choose multiple items)?

□Chemotherapy □Target therapy □Immunotherapy □Surgery

□Radiotherapy □Hormone therapy □Interventional therapy □Other, please specify:___

11 Does the patient have any other chronic illness? □No □Yes, please specify:_____________

12 Please tell me about the patient’s status of ambulation during these days?

□Full □Reduced □Mainly sit or lie □Totally bed bound

13 Please tell me the patient’s activity level during these days?

□Normal activity and work, no evidence of disease

□Normal activity and work, some evidence of disease

□Normal activity with effort, some evidence of disease

□Unable to do normal job or work, significant disease

□Unable to engage in hobbies/ house work, significant disease

□Unable to do any work, extensive disease

□Unable to do most activities, extensive disease

□Unable to do any activity, extensive disease

14 Please tell me how does the patient perform self-care?

□Full □Occasional assistance necessary □Considerable assistance required

□Mainly assistance □Total care

15 How much does the patient eat these days?

□Normal □Normal or reduced □Minimal to sips □Mouth care only

16 How is the patient’s conscious status?

□Full □Full or confusion □Full or drowsy +/- Confusion □Drowsy or coma +/- Confusion

17 How much does the patient know his/her own disease?

□Did not know the diagnosis □Only knew the diagnosis □Knew the diagnosis and the severity

**Part III: Information about family caregiving for the patient**

1 Who is the primary caregiver of the patient during this period?

□Patient’s spouse □Patient’s children □Patient’s parents

□Patient’s siblings □Grandchildren □Others, please specify: ___________

2 The age of the primary caregiver:___________

3 The working status of the primary caregiver:

□Full-time job with no absence □Full-time job with frequent absence

□Part-time job □Retired □Else, please specify:_________

4 How long does the primary caregiver spend on caring for the patient every day since the admission? _________

5 Do you have another one in need of being cared for at home?

□Nobody □Parents □Children

□Grandchildren □Others, please specify: ___________

6 How do you think of the primary caregiver’s health status?

□1=Very poor □2=Poor □3=Moderate □4=Good □5=Very good

7 How do you think of your care burden?

□No burden □Low burden □Moderate burden

□High burden □Very high burden

8 Did you hire a nursing assistant for the patient during the hospitalization? □No □Yes

**Part IV Participants’ preference of healthcare at the end-of-life stage**

1 Have your ever considered choosing hospice care for the patient when he/she was at the end-of-life stage?

□No □Yes □Unable to decide □Never thought about it

□Not aware of hospice care □Refuse to answer this question

2 The reasons for your refusal of hospice care (if you chose “No” for the question above)

□We should try our best to save the patient life

□Choosing hospice care means giving up, the relatives and friend may blame us

□Giving active treatment to the patient is a way to show the filial piety

□The hospice in which the patient has been treated does not have the hospice service

□I do not know of the hospice service

□The hospice care is not helpful

□Other reasons, please specify:__________

3 The reasons for your choice of hospice care (if you chose “Yes” for the question above)

□It could make the patient experience less pain

□It could let the patient have a better quality of life

□It could let the patient avoid unnecessary treatment

□Other reasons, please specify
